# Association of Androgen Excess with Glucose Intolerance in Women with Polycystic Ovary Syndrome

**DOI:** 10.1155/2018/6869705

**Published:** 2018-03-08

**Authors:** Bingjie Zhang, Jing Wang, Shanmei Shen, Jiayi Liu, Jie Sun, Tianwei Gu, Xiao Ye, Dalong Zhu, Yan Bi

**Affiliations:** ^1^Department of Endocrinology, Drum Tower Hospital Affiliated to Nanjing University Medical School, No. 321 Zhongshan Road, Nanjing 210008, China; ^2^Health Manager Center, Drum Tower Hospital Affiliated to Nanjing University Medical School, No. 321 Zhongshan Road, Nanjing 210008, China

## Abstract

Women with polycystic ovary syndrome (PCOS) show high prevalence of glucose intolerance. This study aimed to investigate the association of androgen excess with glucose intolerance in PCOS. A total of 378 women with PCOS participated in the study. Free androgen index (FAI) was selected as indicator of hyperandrogenism. Insulin sensitivity was assessed by 1/homeostasis model assessment of insulin resistance (1/HOMA-IR) and Matsuda insulin sensitivity index (ISI_M_); *β*-cell function was assessed by disposition index (DI). We found that women with glucose intolerance had higher FAI levels compared to women with normal glucose tolerance (NGT) (prediabetes 6.2, T2DM 7.9 versus NGT 5.0, resp.; *p* < 0.001). Furthermore, there was a direct association between FAI levels and frequency of glucose intolerance (OR = 2.480, 95% CI 1.387–4.434), even after adjusting for age, BMI, waist circumference, hypertension, fasting insulin, testosterone, SHBG, and family history of diabetes. In addition, with FAI increase, glycosylated hemoglobin (HbA1c), plasma glucose concentrations, and serum insulin levels increased, while insulin sensitivity and *β*-cell function decreased. Our results suggested that androgen excess indicated by high FAI levels might serve as indicator of glucose intolerance, as it might promote insulin resistance and *β*-cell dysfunction in women with PCOS.

## 1. Introduction

Polycystic ovary syndrome (PCOS) is an endocrine disease affecting about 6–15% of women of reproductive age [1]. It is a heterogeneous disorder characterized by several clinical features: anovulatory infertility, hyperandrogenism, obesity, insulin resistance, and metabolic syndrome [2]. The long-term health consequences of PCOS range from metabolic abnormalities to increased cardiovascular events [3].

Glucose intolerance is a kind of metabolic abnormality that affects about 22.4% of Chinese women with PCOS [4]. A meta-analysis including 35 studies showed that women with PCOS had increased prevalence of glucose intolerance compared with BMI-matched controls [5]. In addition, the annual conversion rate from normal glucose tolerance (NGT) to abnormal glucose tolerance showed being 16% in obese PCOS women and 1–5% in general obese population, respectively [6]. Previous studies have demonstrated that central adiposity, chronic hyperinsulinemia, and use of certain oral contraceptives (OCs) are risk factors for glucose intolerance in PCOS [7].

Androgen excess, associated with higher risk of insulin resistance, metabolic syndrome, and hepatic steatosis, is a defining feature of PCOS [8]. In present clinical practice, serum total testosterone (TT) level is the most common measure for the investigation of hyperandrogenism. Nevertheless, according to certain studies, testosterone evaluation alone may potentially lead to omissions related to androgen excess [9]. Also, there is a controversy between existing studies on relationships between testosterone and glucose intolerance, since some reported higher testosterone levels in PCOS women with either impaired glucose tolerance (IGT) or type 2 diabetes mellitus (T2DM) compared to those with NGT [10], while others found no differences [11]. Sex hormone-binding globulin (SHBG) has been reported as a protein which could bind to testosterone and regulate its clinical action [12], and there is increasing evidence showing that lower circulating levels of SHBG contribute to T2DM and metabolic syndrome in the general population [13]. Free androgen index (FAI) was regarded as having better diagnostic capacity for hyperandrogenism as it considers both the influences of testosterone and SHBG [14]. Nevertheless, there is limited information on the associations between FAI and glucose intolerance in women with PCOS.

Therefore, we conducted a retrospective study to evaluate if androgen excess as indicated by high FAI characterizes a higher prevalence of glucose intolerance, and the potential associations between androgen excess, insulin sensitivity, and islet *β*-cell function.

## 2. Materials and Methods

### 2.1. Subjects

This is a retrospective cross-sectional study with data collection on 378 women with PCOS who visited the Endocrinology Department of Drum Tower Hospital during a period between April 2013 and March 2017. Patients aged between 18 and 40 years, without pregnancy and diabetes history, and those not taking antidiabetic medication or drugs influencing androgen levels for the past 3 months prior to the study were enrolled in this study. The Institutional Review Board of Drum Tower Hospital has approved the study.

Diagnosis of PCOS was done according to 2003 Rotterdam criteria [15], which state that PCOS can be diagnosed if 2 out of the following 3 characteristics are met: chronic oligo- and/or amenorrhea, hyperandrogenism, and ultrasound with polycystic ovaries. Chronic oligo- and/or amenorrhea were defined as menstrual cycle length > 35 days with < 8 menstrual cycles per year. Hyperandrogenism was defined as hirsutism (modified Ferriman-Gallwey score > 8), severe acne, and/or elevated serum testosterone levels (normal range < 2.53 nmol/L). Polycystic ovaries were defined for cases where at least one ovary had 12 or more follicles with a diameter of 2–9 mm and/or increased ovarian volume (≥10 mL) detected by ultrasound imaging. Premature ovarian failure, hyperprolactinemia, hypothyroidism, congenital adrenal hyperplasia, Cushing's syndrome, and adrenal tumors were excluded.

### 2.2. Demographic Data

Standard anthropometric data and medical history were obtained from each subject. Weight and height measurements were performed with patients wearing light clothing. Body mass index (BMI) was determined according to their height and weight. Waist-to-hip ratio (WHR) was calculated based on the ratio between the standing waist and hip circumference. In addition, measurement of blood pressure was conducted twice during rest time, and the average value was recorded. According to the established obesity criterion in China [16], normal weight, overweight, and obesity were defined as 18 kg/m^2^ ≤ BMI < 24 kg/m^2^, 24 kg/m^2^ ≤ BMI < 28 kg/m^2^, and BMI ≥ 28 kg/m^2^, respectively.

### 2.3. Oral Glucose Tolerance Test

All participants received a 75 g oral glucose tolerance test (OGTT) after fasting for at least 10 hours. Subjects were required to ingest a solution which contains 75 g anhydrous glucose in 5 minutes. Venous blood samples were collected during fasting period and 30, 60, and 120 minutes after anhydrous glucose intake.

Based on the results of OGTT, subjects were then classified into three different glucose tolerance statuses recommended by the International Diabetes Federation (IDF) guideline [17]: NGT with fasting plasma glucose (FPG) < 5.6 mmol/L and 2 h postload plasma glucose (2hPG) < 7.8 mmol/L; T2DM with FPG ≥ 7.0 mmol/L and/or 2hPG ≥ 11.1 mmol/L; the rest were classified as prediabetes. Prediabetes and T2DM were both defined as glucose intolerance.

### 2.4. Biochemical Measurements

Biochemical measurements were performed during the 3–5 days after a spontaneous menstruation. Measurements of plasma glucose, total cholesterol, triglycerides, high density lipoprotein cholesterol (HDL-cholesterol), low density lipoprotein cholesterol (LDL-cholesterol), glycosylated hemoglobin (HbA1c), serum insulin, testosterone, and SHBG were performed at Drum Tower Hospital. Plasma glucose was tested using a hexokinase method (TBA-200FR, Tokyo, Japan). Serum insulin was examined using electrochemiluminescent immunoassay (Roche, USA). Serum testosterone, sulfated dehydroepiandrosterone (DHEAS), and SHBG were measured by chemiluminescence analysis (Siemens, Bad Nauheim, Germany).

### 2.5. Calculations

FAI was calculated by the following equation: FAI = serum testosterone (nmol/L)/serum SHBG (nmol/L) × 100. Insulin sensitivity was estimated by 1/homeostasis model assessment of insulin resistance (1/HOMA-IR) index. HOMA-IR was calculated by the following formula: HOMA-IR = FPG (mmol/L) × fasting insulin (uIU/mL)/22.5 [18]. Matsuda insulin sensitivity index (ISI_M_) was further calculated as it reflects a composite estimate of hepatic and muscle insulin sensitivity [19]. Basal insulin release was assessed according to the homeostasis model assessment of insulin secretion (HOMA-*β*) [18]. OGTT induced insulin secretion was calculated based on the ratio between insulin and glucose area under the curve, while the early-phase insulin secretion was estimated by InsAUC30/GluAUC30 and the total insulin secretion was estimated by InsAUC120/GluAUC120, respectively. We further calculated disposition index (DI) as estimation of insulin resistance-adjusted *β*-cell function using the following formula: DI30 = [InsAUC30/GluAUC30]  ×  ISI_M_ and DI120 = [InsAUC120/GluAUC120]  ×  ISI_M_ [20].

### 2.6. Statistical Analyses

Data were presented as mean ± SD or medians (25th–75th interquartile range). Categorical variables were shown as percentage (%). Normal distribution of all continuous variables was tested with the Kolmogorov–Smirnov test. Variables (HbA1c, insulin, triglycerides, testosterone, SHBG, and FAI) were logarithmically transformed before further analysis. Group differences were analyzed using ANOVA test, general linear model analyses (age- and BMI-adjusted), or *χ*^2^ test. The following Bonferroni method was used for post hoc pairwise comparison. Multivariate logistic regression analysis was performed to assess the association between FAI and the presence of glucose intolerance (including prediabetes and T2DM), adjusted for confounding factors (BMI, age, waist circumference, hypertension, fasting insulin, testosterone, SHBG, and family history of diabetes). Relationships between FAI and the indexes of insulin sensitivity and *β*-cell function were established using Spearman correlation analysis (adjusting for age and BMI). All statistical analyses were performed using SPSS 22.0 software (SPSS Inc., Chicago, IL, USA).

## 3. Results

### 3.1. Characteristics of Participants

A total of 378 PCOS women were included in the analysis. Subjects' mean age was 27.8 ± 4.4 years. Categorized by BMI, 87.3% (330/378) subjects were overweight or obese, while the patients with normal weight accounted for 12.7% (48/378). Based on the oral glucose tolerance test, 59.8% (226 of 378) of the subjects had NGT, 31.5% (119 of 378) had prediabetes, and 8.7% (33 of 378) had T2DM.

Clinical and laboratory characteristics of the subjects are shown in Table 1. No difference in prevalence of oligomenorrhea, hyperandrogenism, and polycystic ovaries was observed in women with PCOS with different glucose metabolism statuses. As expected, subjects included in the prediabetes and T2DM groups had significantly higher BMI, waist circumference, WHR, fasting glucose, and fasting serum insulin levels compared to those in the NGT group. Furthermore, higher triglycerides levels were observed in women with prediabetes compared to women with NGT, while similar levels of other lipid profiles (total cholesterol, HDL-cholesterol, and LDL-cholesterol) were detected in all groups. Moreover, no difference in the frequency of family history of diabetes was observed; however, hypertension was more frequent in women with glucose intolerance compared to those with NGT (*p* < 0.001).

### 3.2. Androgen among Different Glucose Metabolism Status

**Figure 1:**
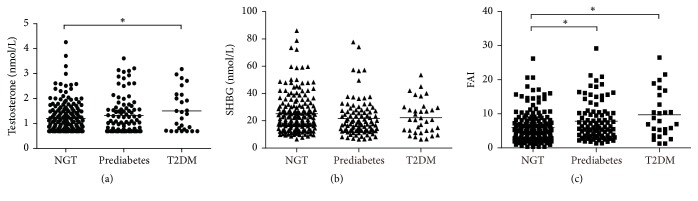
Testosterone, SHBG, and FAI levels in different glucose tolerance status in women with PCOS. General linear model adjusted based on age and BMI was used for comparison between groups. (a) Testosterone levels in different glucose tolerance status (*p* = 0.020). (b) SHBG levels in different glucose tolerance status (*p* = 0.090). (c) FAI levels in different glucose tolerance status (*p* < 0.001). ^*∗*^*p* < 0.015.

Significant different levels of total testosterone were found between subjects with diabetes and those with NGT, while DHEAS levels did not differ among the three groups (*p* = 0.672). Compared with the NGT group, PCOS women with prediabetes had lower serum SHBG concentration. Furthermore, FAI was significantly higher in subjects with glucose intolerance compared to subjects with NGT (prediabetes 6.2, T2DM 7.9 versus NGT 5.0, resp.; *p* < 0.001) (Table 1). In addition, after adjusting for age and BMI, difference in SHBG lost the significance (*p* = 0.090) (Figure 1(b)), while differences in testosterone and FAI remained significant (*p* = 0.020 and *p* < 0.001, resp.) (Figures 1(a) and 1(c)).

### 3.3. Risk Factors for Glucose Intolerance

In regression analysis, age, BMI, waist circumference, hypertension, fasting insulin, testosterone, SHBG, FAI, and family history of diabetes were significant factors associated with glucose intolerance. After multivariate logistic regression analysis, only age (OR = 1.159, 95% CI 1.044–1.288), hypertension (OR = 2.984, 95% CI 1.111–8.016), and FAI (OR = 1.166, 95% CI 1.069–1.272) showed being independent risk factors for glucose intolerance in women with PCOS. Additionally, FAI was analyzed as a categorical variable divided into tertiles. The highest tertile of FAI showed significantly higher rate of glucose intolerance compared to the lowest tertile (OR = 2.480, 95% CI 1.387–4.434) (Table 2).

### 3.4. Characteristics of Women with PCOS among FAI Tertiles

Increasing tertiles of FAI were accompanied with higher prevalence of glucose intolerance (31.3%, 37.8%, and 53.1%, resp.) (Table 3). No differences in waist circumference and WHR were found among the three groups. Moreover, with increasing tertiles of FAI, HbA1c, and plasma glucose concentrations (fasting, 30 min, 1 h, and 2 h after glucose intake) increased as well. Additionally, basal and OGTT stimulated insulin secretion showed similar trends to FAI tertiles (all *p* ≤ 0.001).

### 3.5. Associations of FAI with Indexes of Insulin Sensitivity and *β*-Cell Function

Notably, FAI was negatively related to 1/HOMA-IR and ISI_M_ (*r* = −0.413, *p* < 0.001; *r* = −0.407, *p* < 0.001), while it was positively related to HOMA-*β*, InsAUC30/GluAUC30, and InsAUC120/GluAUC120 (*r* = 0.337, *p* < 0.001; *r* = 0.221, *p* < 0.001; *r* = 0.236, *p* < 0.001). Moreover, inverse relationships were found between FAI and DI30 and DI120 (*r* = −0.154, *p* = 0.012; *r* = −0.189, *p* = 0.002). After age and BMI adjustment, associations between FAI and all the indexes remained significant (Table 4).

**Figure 2:**
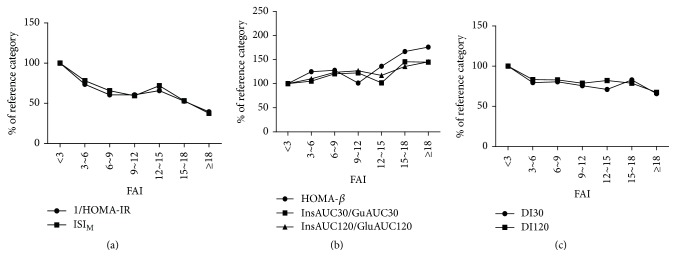
Indexes of insulin sensitivity (a), insulin release (b), and *β*-cell function (c) across categories of FAI. Calculations were adjusted for age and BMI using general linear model. Data are shown as percentage of each point compared to the reference category. One-way analysis of variance showed significant trends for all the indexes (1/HOMA-IR, *p* < 0.001; ISI_M_, *p* < 0.001; HOMA-*β*, *p* < 0.001; InsAUC30/GluAUC30, *p* = 0.004; InsAUC120/GluAUC120, *p* = 0.009; DI30, *p* = 0.004; DI120, *p* < 0.001).

Furthermore, according to every three increments in FAI, subjects were divided into several categories. Briefly, subjects with FAI < 3 were regarded as the reference category. With FAI increased, insulin sensitivity (1/HOMA-IR and ISI_M_) decreased (Figure 2(a)), while insulin secretion (HOMA-*β*, InsAUC30/GluAUC30, and InsAUC120/GluAUC120) showed a gradually upward trend (Figure 2(b)). In addition, *β*-cell function adjusted for insulin resistance decreased (−22% in DI30 and −19% in DI120) in subjects with FAI 3–18 and significantly reduced (−34% in DI30 and −33% in DI120) in subjects with FAI ≥ 18, compared to the reference category (Figure 2(c)).

## 4. Discussion

In this study, we found that androgen excess, as indicated by high FAI levels, was associated with higher rates of glucose intolerance, and FAI could be regarded as indicator of insulin resistance and *β*-cell dysfunction in PCOS women.

PCOS is one of the most common disorders of reproductive-aged women and is characterized by a high prevalence of metabolic abnormalities. Women with PCOS showed increased risk of glucose intolerance compared with BMI-matched controls [5]. Previous studies have reported that obesity, hyperinsulinemia, and use of oral contraceptives are risk factors for glucose intolerance in PCOS. Our study focused on the role of androgen excess, which is a defining feature of PCOS. In this study, we found an association between elevated FAI and glucose intolerance. Women with PCOS in the highest tertile of FAI had a 1.48-fold increased prevalence of glucose intolerance and elevated FAI was identified as an independent risk factor.

Previous studies reported that PCOS women with IGT or T2DM had higher testosterone levels compared to those with NGT [4, 7, 10], in accordance with our findings. However, there is a controversy as others found no differences [11]. The probable reason is that most studies selected just one kind of androgens (such as total testosterone/androstenedione) as the indicator of androgen excess, which may lead to some omissions as PCOS women always show different features of biochemical hyperandrogenism [9]. Free androgen index, determined by serum total testosterone and SHBG levels, has been confirmed to be closely related to all kinds of androgens and with little influence from metabolic components [21, 22]. Therefore, we took FAI as the indicator of androgen excess. Previous studies demonstrated that FAI was associated with insulin resistance, blood pressure progression, and vascular aging in the general population, indicating its important role in cardiovascular progression and metabolic diseases [23, 24]. An investigation involving a Mediterranean population revealed significantly higher levels of FAI in PCOS patients with IGT or T2DM [25], which is in favor of our findings. However, with limited sample size, this study did not further investigate the association of high FAI levels with glucose intolerance. Another study conducted in Chinese population identified an association between FAI and metabolic syndrome [26]. In our research, we demonstrated higher FAI in patients with glucose intolerance and moreover we found FAI to be an independent risk factor for high prevalence of glucose intolerance in women with PCOS.

In the present study, we also found that in PCOS population FAI was associated with both insulin sensitivity and *β*-cell function. As FAI increased, insulin sensitivity and *β*-cell function decreased. An imbalance between insulin sensitivity and insulin secretion is the key pathophysiological mechanism in the progression of glucose intolerance. Insulin resistance in peripheral tissues decreases insulin-stimulated glucose uptake. When intense loss of *β*-cell function occurs, insulin secretion becomes insufficient, which eventually leads to dysglycemia [27]. Previous studies have reported similar findings, especially with regard to the influence of androgen excess on insulin resistance. Researchers found that the classic phenotype of PCOS was independently associated with insulin resistance, whereas the normoandrogenic phenotype was not, indicating the significant influence of hyperandrogenism on insulin resistance [28]. Also, a cross-sectional population-based study including 833 PCOS patients of reproductive age yielded a strong association between FAI and insulin resistance [26]. However, to our knowledge, the present study is the first to report on elevated FAI and its ability to aggravate the occurrence of glucose intolerance and *β*-cell dysfunction. These findings have preventive and therapeutic implications as they suggest that PCOS women with high FAI levels should pay particular attention to diabetes screening, as they are at increased risk of glucose intolerance compared to PCOS patients with low FAI levels.

The mechanisms could be partially explained by excess androgens in female promoting hepatic insulin resistance by repressing NF-kB induced protein-tyrosine phosphatase 1B (PTP1B) [29], reducing skeletal glucose uptake by decreasing activation of serine/threonine-specific protein kinase AKT and glucose transporter type 4 (GLUT4) expressions [30]. Furthermore, systemic oxidative stress produced by excess androgen receptor (AR) activation as well as mononuclear cell- (MNC-) derived oxidative stress may do harm to the *β*-cell dysfunction [31, 32]. Meanwhile, hyperandrogenemia would also impair insulin secretion through disruption of *β*-cell mitochondrial function [33], finally resulting in higher blood glucose levels. In addition, previous data also support significant influence of hyperinsulinemia on androgens. In vitro, insulin may stimulate LH-dependent ovarian androgen secretion in theca cells and suppress liver SHBG synthesis, thus leading to increased free androgen levels [34, 35]. Meanwhile, in PCOS models whose insulin receptors in ovarian theca cells have been deleted, hyperandrogenism is corrected to a large extent, indicating that hyperinsulinemia plays a major role in triggering hyperandrogenemia [36].

There were some limitations in our study. Firstly, the present study was observational without long-term follow-up. The predictive potential of FAI in relation to glucose intolerance was not investigated over a certain time period. Secondly, we did not evaluate other androgens like androstenedione which is also proved to be a sensitive indicator of PCOS-related androgen excess; however, FAI has been closely related to all kinds of androgens and confirmed as a good marker of biochemical hyperandrogenism with little influence from metabolic components [21, 22]. Thirdly, we used immunoassay to measure androgen levels which might not be precise enough for measuring low hormone levels [37], thus leading to increased measurement error and diluting potential risk associations. Future studies should rely on more highly sensitive methods based on LC-MS/MS and detect more kinds of androgens. Finally, instead of the golden standard for insulin sensitivity evaluation, hyperinsulinemic euglycemic clamp, we used 1/HOMA-IR and ISI_M_ which are simple and reliable measurements but might not be accurate enough.

The present study demonstrated that elevated FAI was significantly associated with higher rates of glucose intolerance in women with PCOS. These mechanisms could be partially explained by severe insulin resistance and damaged islet *β*-cell function. Further studies with long-term follow-up are needed to evaluate the potential effect of reducing hyperandrogenism on glucose metabolism and to explore the exact mechanisms.

## 5. Conclusion

In summary, androgen excess indicated by high FAI levels might serve as indicator of glucose intolerance by influencing insulin resistance and islet *β*-cell function in women with PCOS.

## Figures and Tables

**Table 1:** Characteristics of subjects based on different glucose tolerance status.

	NGT	Prediabetes	T2DM	*p*
*n*	226	119	33	-
Age, y	27.1 ± 4.5	29.0 ± 4.1^*∗*^	28.5 ± 4.4	0.001
Oligomenorrhea, %	167 (73.9)	79 (66.4)	25 (75.8)	0.259
Hyperandrogenism, %	145 (64.2)	72 (60.5)	21 (63.6)	0.834
Polycystic ovaries, %	190 (84.1)	97 (81.5)	29 (87.9)	0.717
BMI, kg/m^2^	29.3 ± 5.5	30.6 ± 4.3^*∗*^	31.5 ± 4.4^*∗*^	0.012
Waist circumference, cm	90.6 ± 14.4	96.1 ± 12.0^*∗*^	97.2 ± 8.8^*∗*^	0.014
Waist-to-hip ratio	0.88 ± 0.08	0.91 ± 0.06^*∗*^	0.92 ± 0.09^*∗*^	0.014
Systolic BP, mmHg	120.3 ± 13.7	125.4 ± 14.1^*∗*^	134.6 ± 17.7^*∗*#^	<0.001
Diastolic BP, mmHg	78.7 ± 11.7	83.5 ± 12.6^*∗*^	91.3 ± 13.3^*∗*#^	<0.001
Hypertension, %	32 (14.2)	32 (26.9)^*∗*^	15 (45.5)^*∗*^	<0.001
Fasting glucose, mmol/L	4.9 ± 0.4	5.4 ± 0.5^*∗*^	6.4 ± 1.0^*∗*#^	<0.001
Fasting insulin, uIU/mL	17.8 (12.3–26.1)	21.8 (16.3–31.4)^*∗*^	25.0 (20.3–38.5)^*∗*^	<0.001
Family history of diabetes, %	36 (15.9)	33 (27.7)	6 (18.2)	0.056
Triglycerides, mmol/L	1.5 (1.1–2.2)	1.7 (1.3–2.4)^*∗*^	1.8 (1.4–2.4)	0.028
Total cholesterol, mmol/L	4.7 ± 0.9	4.8 ± 1.1	4.7 ± 0.7	0.801
HDL-cholesterol, mmol/L	1.2 ± 0.3	1.1 ± 0.5	1.0 ± 0.2	0.296
LDL-cholesterol, mmol/L	2.7 ± 0.7	2.7 ± 0.9	2.8 ± 0.7	0.659
Testosterone, nmol/L	1.0 (0.7–1.5)	1.1 (0.7–1.6)	1.4 (0.7–2.2)^*∗*^	0.033
DHEAS, ug/dL	235.9 ± 109.0	235.9 ± 104.9	216.6 ± 106.4	0.672
SHBG, nmol/L	22.1 (17.0–31.6)	17.6 (12.9–26.8)^*∗*^	18.2 (11.2–26.9)	0.028
FAI	5.0 (2.8–7.6)	6.2 (3.6–10.3)^*∗*^	7.9 (4.4–15.5)^*∗*^	<0.001

NGT, normal glucose tolerance; T2DM, type 2 diabetes mellitus; BMI, body mass index; BP, blood pressure; DHEAS, sulfated dehydroepiandrosterone; SHBG, sex hormone-binding globulin; FAI, free androgen index. Data are presented as mean ± SD, median (25th–75th interquartile range), or *n* (%). ^*∗*^*p* < 0.015 versus NGT group; ^#^*p* < 0.015 versus prediabetes group.

**Table 2:** Odds ratio and 95% CI of glucose intolerance according to FAI levels.

FAI	Odds ratio (95% CI)
Continuous variable per unit increment	1.166 (1.069–1.272)^*∗*^
Tertile	
I	1.000 [reference]
II	1.331 (0.738–2.398)
III	2.480 (1.387–4.434)^*∗*^
*p* for trend	0.002

CI, confidence interval; FAI, free androgen index. ^*∗*^*p* < 0.05.

**Table 3:** Anthropometric and clinical characteristics of women with PCOS according to tertiles of FAI.

	FAI < 3.77	3.77 ≤ FAI < 7.44	FAI ≥ 7.44	*p*
*n*	99	98	98	-
Age, y	29.0 ± 4.8	27.9 ± 4.4	27.0 ± 4.0^*∗*^	0.007
BMI, kg/m^2^	28.6 ± 5.3	29.7 ± 4.6	30.8 ± 5.0^*∗*^	0.011
Waist circumference, cm	88.4 ± 16.6	91.0 ± 13.0	95.5 ± 13.6	0.055
Waist-to-hip ratio	0.88 ± 0.09	0.89 ± 0.07	0.91 ± 0.07	0.276
HbA1c, %	5.3 (5.0–5.5)	5.4 (5.1–5.6)	5.5 (5.3–5.7)^*∗*^	0.045
Fasting glucose, mmol/L	5.1 ± 0.6	5.2 ± 0.7	5.3 ± 0.8	0.069
30 min glucose, mmol/L	8.2 ± 1.3	8.7 ± 1.4	9.1 ± 1.7^*∗*^	<0.001
1 h glucose, mmol/L	8.7 ± 2.1	9.0 ± 2.3	10.0 ± 2.4^*∗*#^	0.001
2 h glucose, mmol/L	7.4 ± 2.0	7.6 ± 2.0	8.2 ± 2.2^*∗*^	0.029
Fasting insulin, uIU/mL	15.2 (9.0–20.9)	19.6 (14.6–28.1)^*∗*^	24.9 (17.7–35.4)^*∗*#^	<0.001
30 min insulin, uIU/mL	106.9 (70.2–159.0)	125.0 (87.9–175.6)	153.7 (97.1–221.9)^*∗*^	0.001
1 h insulin, uIU/mL	105.6 (76.1–151.8)	137.3 (89.5–225.7)^*∗*^	186.7 (124.5–275.1)^*∗*#^	<0.001
2 h insulin, uIU/mL	99.2 (70.8–166.6)	115.4 (76.2–208.0)	171.2 (97.5–276.3)^*∗*#^	0.001
Glucose intolerance, %	31 (31.3)	37 (37.8)	52 (53.1)^*∗*^	0.006

PCOS, polycystic ovary syndrome; FAI, free androgen index; BMI, body mass index. Data are presented as mean ± SD, median (25th–75th interquartile range), or *n* (%). ^*∗*^*p* < 0.015 versus FAI < 3.77 group; ^#^*p* < 0.015 versus 3.77 ≤ FAI < 7.44 group.

**Table 4:** Correlation between FAI and insulin sensitivity and *β*-cell function.

	FAI			
	*r*	*p* value	*R*	*p* value (age- and BMI- adjusted)
1/HOMA-IR	−0.413	<0.001	−0.288	<0.001
ISI_M_	−0.407	<0.001	−0.297	<0.001
HOMA-*β*	0.337	<0.001	0.226	<0.001
InsAUC30/GluAUC30	0.221	<0.001	0.140	0.023
InsAUC120/GluAUC120	0.236	<0.001	0.141	0.022
DI30	−0.154	0.012	−0.176	0.004
DI120	−0.189	0.002	−0.205	0.001
